# Psychometric Properties of the French Version of the Physical Literacy Self-Description (PLSD) Questionnaire Among Children

**DOI:** 10.3390/bs16091672

**Published:** 2026-09-17

**Authors:** Christophe Maïano, Alexandre J. S. Morin, Johanne April, Olivier Hue, Olivier Rey

**Affiliations:** 1 Department of Psychoeducation and Psychology, Université du Québec en Outaouais, Saint-Jérôme, QC J7Z 0B7, Canada; 2 Cyberpsychology Laboratory, Department of Psychoeducation and Psychology, Université du Québec en Outaouais, Gatineau, QC J8X 3X7, Canada; 3 Substantive-Methodological Synergy Research Laboratory, Department of Psychology, Concordia University, Montréal, QC H4B 1R6, Canada; alexandre.morin@concordia.ca; 4 Optentia Research Unit, North-West University, P.O. Box 1174, Vanderbijlpark 1900, South Africa; 5 Department of Educational Sciences, Université du Québec en Outaouais, Saint-Jérôme, QC J7Z 0B7, Canada; johanne.april@uqo.ca; 6 Department of Physical Activity Sciences, Université du Québec à Trois-Rivières, Trois-Rivières, QC G9A 5H7, Canada; olivier.hue@uqtr.ca; 7 ISM, CNRS, Aix Marseille University, 13288 Marseille Cedex 09, France; olivier.rey@univ-amu.fr

**Keywords:** physical literacy, French, cross-sectional validation study, exploratory factor analyses, measurement invariance, differential item functioning, physical activity

## Abstract

This study sought to document the psychometric properties of scores on the French Version of the Physical Literacy Self-Description questionnaire. A sample of 191 French-speaking Canadian and French children, aged 5–12 years, participated in this study. Results from an exploratory factor analytic solution supported a two-factor solution encompassing (a) perceived confidence and value; and (b) perceived skills competence. Subsequent analyses supported the weak, strong and strict invariance of this solution as a function of sex, and its weak, strong, and partial strict invariance (one item was less reliable in France) across countries. The results also revealed meaningful differences in terms of latent variances (showing more inter-individual variability in Canada and among boys) and covariances (showing stronger correlations in France and among boys) across grouping variables. Results further revealed a lack of differential item functioning as a function of age and time spent in moderate to vigorous physical activities, but minimal evidence of differential item functioning as a function of body mass index (on two items). Moreover, Canadian children scored higher on perceived confidence and value and lower on perceived skills competence, while younger children scored higher on perceived skills competence. Likewise, children spending more time in moderate physical activity scored higher in the perceived confidence and value factor, while those spending more time in vigorous physical activity scored higher on the perceived skills competence factor. Finally, results supported the convergent validity of scores obtained on the French Physical Literacy Self-Description questionnaire in relation to parental ratings of children’s overall, cognitive, and motor physical literacy.

## 1. Introduction

Across the past two decades, the concept of physical literacy among school-aged children has gained a growing interest among researchers and practitioners from the fields of physical education, physical activity and sport sciences (Barnett et al., 2023; Edwards et al., 2017). The International Physical Literacy Association (2017) defines physical literacy as “*the motivation, confidence, physical competence, knowledge and understanding to value and take responsibility for engagement in physical activities for life*” (Physical literacy section, para. 1). This multidimensional concept thus encompasses the affective (e.g., motivation and confidence), behavioral (engagement), cognitive (knowledge) and physical (competence) capabilities required to be physically active over the lifespan (Tremblay et al., 2018).

This growing interest has led to the development of various instruments designed to assess the physical literacy of school-aged children ranging from early primary school to secondary school (Barnett et al., 2023; Grauduszus et al., 2023). Many of these tools have been developed in Canada^1^ (*Canadian Assessment of Physical Literacy*: Healthy Active Living and Obesity Research Group, 2017; *Passport for Life*: Physical and Health Education Canada, 2013; *PLAY tools: basic, coach, fun, parents, self, and pre-play*: Sport for Life, 2022a, 2022b, 2022c, 2022d, 2022e, 2022f), are freely available in English and in French, and encompass an objective (i.e., fitness and movement skills) and subjective (i.e., self-report questionnaire) assessment of physical literacy (Barnett et al., 2023; Grauduszus et al., 2023). Among those Canadian tools available in both languages, the *PLAY tools* (Sport for Life, 2022a, 2022b, 2022c, 2022d, 2022e, 2022f) are particularly promising. This suite of assessment tools includes measures that can be completed by trained professionals, parents, coaches, and other practitioners, while also incorporating the Physical Literacy Self-Description (PLSD), a brief self-report questionnaire designed for children. The inclusion of a short self-report measure is especially valuable, as tools such as the PLSD can be easily administered in school and community settings, providing a practical and accessible means of assessing children’s perceptions of their physical literacy and supporting program evaluation and monitoring initiatives. Current evidence also supports the reliability and validity of scores on the PLSD (Caldwell et al., 2021; Gilic et al., 2022; Jefferies et al., 2021; Kleis et al., 2022; Lininger & Root, 2024; Vuletic et al., 2023), although limitations remain, thereby reinforcing the need for additional psychometric research.

Despite the availability of several Canadian physical literacy assessment tools in both English and French, evidence supporting their psychometric properties has been primarily obtained among English-speaking samples. These psychometric properties do not automatically generalize to French-speaking populations. This issue is particularly important for the PLSD, given that the scoring is highly dependent on children’s understanding of the content of the items and of the response scale. Therefore, it is important to examine whether psychometric properties obtained among English-speaking samples can generalize to French-speaking children. Such evidence will favor the use of French-speaking samples of children in Canada and France, and cross-cultural physical literacy research using the PLSD.

### 1.1. The Physical Literacy Self-Description Questionnaire

The PLSD (Sport for Life, 2022d) is a 12-item self-report instrument designed to assess children’s beliefs of their ability to successfully engage in physical activity across multiple dimensions of physical literacy. Grounded in the multidimensional nature of physical literacy, the PLSD includes items related to confidence, values, and technical competence, reflecting distinct affective and physical aspects of the construct. From a theoretical perspective, confidence and values are associated with the emotional and evaluative dimensions of participation in physical activity, including children’s beliefs in their capacity to be physically active and the importance they attribute to an active lifestyle. In contrast, technical competence relates more closely to children’s perceptions of their motor proficiency and ability to successfully perform movement-related tasks. However, the extent to which these elements form distinct dimensions within the PLSD remains unclear. Consequently, further examination of the instrument’s latent structure is warranted.

Each item is rated on a four-point scale (0—not true at all, 1—not usually true, 2—true, and 3—very true). Item scores are summed to produce a single overall physical literacy score. Although this scoring approach provides a concise indicator of perceived physical literacy, it assumes a unidimensional structure and does not account for the potentially multidimensional nature of the construct. Therefore, factor analytic investigation is needed to determine how the PLSD items are organized and whether a multidimensional representation provides a more accurate interpretation of children’s responses.

Since the development of the PLSD, some studies have examined the psychometric properties of the PLSD (e.g., age-related differential item functioning and convergent validity) among participants from Canada (Jefferies et al., 2021), the United States (US; Kleis et al., 2022; Lininger & Root, 2024) and South Eastern Europe (i.e., Croatia, Bosnia-Herzegovina, and Montenegro; Gilic et al., 2022; Vuletic et al., 2023). However, to our knowledge, only two of those studies have examined the factor validity and reliability of scores obtained on the English language version of the PLSD among samples of US adolescents (14–18 years; Lininger & Root, 2024) and emerging adults (18–25 years; Kleis et al., 2022). These studies thus ignore the age range for which the PLSD was specifically designed (primary school children, so roughly 5–6 years old to 11–12 years old), in addition to providing little information on the companion French version.

In a sample of 245 US emerging adults, Kleis et al. (2022) relied on a principal component analysis (PCA; i.e., a formative model ill-suited to reflective constructs such as physical literacy; Kahn, 2006; Preacher & MacCallum, 2003) to investigate the structure of PLSD scores. Their results revealed two components, albeit the first one (i.e., youth’s perceptions of “*confidence and/or knowledge of skills relating to physical activity*”) included 10 of the 12 PLSD items, whereas the second one included only two items (i.e., perceptions of “*values and/motivation relating to physical activity*”). This ill-balanced separation of items is consistent with the nature of principal component analysis, which seeks to extract a large primary component explaining a maximum of variance, before considering smaller components. Interestingly, despite providing evidence of a two-component structure, the authors only report scale score reliability for the complete instrument (α = 0.89).

For their part, Lininger and Root (2024) relied on exploratory factor analysis (EFA) with maximum likelihood estimation among a sample of 151 US adolescents (14–18 years) who completed the PLSD. After excluding one item, their results revealed a three-factor structure encompassing: (a) *perceived confidence* (four items); (b) *perceived skill ability* (five items); and (c) *perceived value* (two items). This third factor matches the content of Kleis et al.’s (2022) second factor. Finally, the composite reliability of scores obtained on these factors was modest-to-acceptable (ω = 0.69 to 0.78).

### 1.2. The Present Study

Despite their relevance, these previous studies present relatively important limitations. First, there is little evidence about the factor validity and reliability of scores obtained on the PLSD among children, even though primary school children are the main target of this instrument. Second, current research provides inconsistent evidence about the PLSD factor structure. Moreover, the reliance on distinct types of analyses, more (EFA) or less (PCA) relevant to their objectives, makes it hard to contrast the results obtained in these previous studies (Kleis et al., 2022; Lininger & Root, 2024).

Third, there is no evidence of measurement invariance and/or lack of differential item functioning in any of the studies examining the factor validity of scores obtained on the PLSD. This lack of knowledge calls into question the possibility of using this instrument to conduct unbiased comparisons of subsamples of children differing in terms of characteristics commonly examined in physical literacy research (e.g., age, sex, body mass index, and physical activity level).

Fourth, to our knowledge, no previous study examined the factor validity and reliability of scores obtained on the PLSD, together with their potential measurement invariance and/or differential item functioning across French-speaking participants. This lack of knowledge calls into question the relevance of using the French version of this instrument in the context of cross-cultural (or even cross-national in Canada) studies on physical literacy involving French-speaking participants. Practically speaking, addressing this issue will greatly facilitate comparison of physical literacy among European (e.g., Belgium, France, and Switzerland), American (Canada, Haiti, and French Guiana), and African (e.g., Algeria, Cameroon, Morocco, and Tunisia) populations.

The main objective of the present study was to address these limitations by examining the psychometric properties of the French version of the PLSD among French-speaking children recruited in Canada and in France. First, given the lack of clarity regarding the factor structure of the PLSD, an EFA will first be conducted to identify the most appropriate factor solution and to examine its reliability. Subsequently, all additional psychometric analyses will be conducted based on the retained factor structure. More specifically, the measurement invariance of the selected factor solution will be examined as a function of the children’s sex (girls vs. boys) and country (Canada vs. France). Third, the presence of differential item functioning of PLSD ratings as a function of age, body mass index (BMI), and physical activity (moderate to vigorous intensity) will be formally investigated. Finally, the convergent validity of scores on the French version of the PLSD will be examined in relation to parental measures of physical literacy (overall physical literacy and cognitive and motor competence dimensions of physical literacy) from the companion *PLAYparent* instruments.

## 2. Method

### 2.1. Participants

This study relies on a convenience sample of 191 French-speaking children (50.8% boys; aged 5 to 12 years; *M_age_* = 8.62 years, *SD_age_* = 1.88; BMI: 10.54 to 36.28 kg/m^2^, *M_BMI_* = 16.52, *SD_BMI_* = 3.21) recruited in elementary schools (96%) or via community organizations (4%) located in Canada (*N* = 118; 53.4% boys; *M_age_* = 9.03 years; *M_BMI_* = 17.87 kg/m^2^) and France (*N* = 73; 46.6% boys; *M_age_* = 7.97 years; *M_BMI_* = 15.96 kg/m^2^). Each week (Monday to Friday), these children spent on average 295.88 min (Canadian: *M* = 345.88; French: *M* = 272.59) in physical activities of moderate intensity and 109.79 min (Canadian: *M* = 102.79; French: *M* = 113.05) in physical activities of vigorous intensity. Children were not eligible to participate if they displayed characteristics likely to bias their responses to, or limit their ability to respond to, the PLSD (i.e., developmental delay, neurological disorder, sensory or physical disabilities, or mobility assistance).

### 2.2. Measures

#### 2.2.1. Children’s Characteristics

Children’s sex, age, height and weight were reported by parents. Children’s height and weight were used to estimate their BMI (weight/height^2^) expressed in kg/m^2^.

#### 2.2.2. Children’s Physical Literacy

The official French version of the children’s PLSD developed by Sport for Life (2022d) was used to assess children’s physical literacy. This questionnaire comprises 12 items (e.g., *It doesn’t take me long to learn new skills, sports or activities*) that children answer using a 4-point scale ranging from 0 (*not true at all*; French: *pas du tout vrai*) to 3 (*very true*; French: *tout à fait vrai*).

#### 2.2.3. Parental Assessment of Children’s Overall Physical Literacy

The French version of the physical literacy visual analog scale (VAS) section from the *PLAY*parent workbook (Sport for Life, 2022e) was used to obtain parental assessments of their child’s overall level of physical literacy. First, a definition of physical literacy was provided to parents who were then invited to score *how their child’s physical literacy compares to someone with perfect physical literacy* (Sport for Life, 2022e) by placing a mark on a 100 mm VAS ranging from 0% (*not physically literate*; French: *aucune littératie physique*) to 100% (*perfect physical literacy*; French: *littératie physique parfaite*).

#### 2.2.4. Parental Assessment of the Cognitive and Motor Competence Dimensions of Children’s Physical Literacy

The cognitive and motor competence dimensions of children’s physical literacy were obtained from parents using 15 French items from the *PLAY*parent workbook (Sport for Life, 2022e). The cognitive dimension comprises six items measuring parents’ assessment of their child’s confidence (e.g., *confidence to participate in physical activity*), motivation (e.g., *motivation to participate in physical activity and sport*) and comprehension (e.g., *knowledge related to healthy physical activity*) in physical activity and sport. The motor competence dimension comprises nine items measuring parents’ assessment of their child’s locomotor (e.g., ability to balance during movement) and object control (e.g., ability to use feet to kick or move objects) competencies. Parents rated each item using a 3-point scale ranging from 0 (low; French: *faible*) to 2 (high; French: *élevé*). In the present study, McDonald’s ω coefficients (McDonald, 1970) for the cognitive and motor competence dimensions of physical literacy were 0.745 and 0.810, respectively.

#### 2.2.5. Time Spent in Moderate-to-Vigorous Physical Activities

Parents were invited to complete the French proxy-report version of the Children’s Leisure Activities Study Survey (CLASS; Telford et al., 2004). Permission to develop a French adaptation of the CLASS in French was granted in September 2021. The translation process followed established cross-cultural adaptation guidelines using a forward and backward translation procedure (Hambleton, 2005). Initially, a professional bilingual translator who had no prior familiarity with the CLASS translated the questionnaire from English into French. The resulting French version was subsequently translated back into English by a second independent bilingual translator who was blinded to the original questionnaire. The equivalence of the source and back-translated versions was then evaluated through a review process. Differences identified between versions were discussed and resolved by an expert committee composed of the two translators and four members of the research team.

The proxy-report version of the CLASS parents includes a list of 30 physical activities. For each physical activity parents were asked to indicate whether their child engages or not in this physical activity (yes or no) during a typical week (from Monday to Friday during the current school term). Parents reporting that their child was engaging in this physical activity were asked to report the frequency (number of times) and the total duration (in minutes or hours) during a typical week. As recommended by Telford et al. (2004), ratings were converted to minutes per week (Monday to Friday) spent in moderate-to-vigorous physical activities (MVPA).

### 2.3. Procedures

Authorization to conduct the study was obtained from the university research ethics committee of the first (#2022-2068) and last authors’ institutions (#2023-06-15-008). Authorizations were first obtained from school boards and principals. Participants were recruited in six elementary schools that agreed to participate, or in the community via sport and leisure organizations and an advertisement in a local newspaper. No compensation was offered in France, whereas Canadian participants were eligible to win one out of 5 gift certificates ($20 CAD).

Informed consent for participation was obtained from the parents or legal guardians of all children involved in the study, either through paper-based or electronic procedures. For parents recruited through schools, a digital and printed consent form and information letter were sent. Parents who completed the paper version returned the signed document to the school, from where it was collected by a member of the research team. Electronic consent forms, in contrast, were submitted directly to the researchers. For parents of children recruited in the community, the information letter and link to access the online consent form were posted on social media or sent by emails by sport and leisure organizations. The completed online consent form was directly received by the research team. Consenting parents were then invited by email to access an online survey (hosted on LimeSurvey) about their children.

Once parental/legal consent was obtained, children were met individually at school (93.2% in Canada and 100% in France) or via Zoom (6.8% in Canada only) by a member of the research team. The aim of this meeting was to present the objectives and procedures of the study and to obtain the child’s verbal assent to participate (or not) in the study. Researchers followed a standardized administration protocol across both in-person and Zoom assessments to ensure consistency in data collection procedures. Prior to data collection, all members of the research team received training on participant recruitment, questionnaire administration, and response recording. In addition, a meeting was held between countries to ensure adherence to the standardized protocol. Children that verbally agreed to participate were then invited by a member of the research team to complete the PLSD. The PLSD instructions and items were read aloud by the interviewer and children were asked to answer independently. To minimize interviewer influence, members of the research team were instructed not to provide feedback, interpretations, or guidance regarding item content or response selection beyond repeating the standardized instructions when needed. For each item, children were asked to indicate their response, which was subsequently recorded by the interviewer. Completion of the PLSD generally required less than 10 min. Data collection took place from April 2023 to May 2024.

### 2.4. Analyses

Given the ordinal nature and asymmetric thresholds of the response scales used in this study (Finney & DiStefano, 2013), analyses were performed using Mplus 8.11 (Muthén & Muthén, 2024) robust weighted least squares with mean and variance adjustment (WLSMV) estimator. There were no missing responses in this dataset. Exploratory factor analyses were estimated using an oblique Geomin rotation procedure with an epsilon value of 0.5 to maximally reduce factor correlations (Marsh et al., 2009; Marsh et al., 2013) to identify the optimal factor structure underlying responses to the PLSD. A parallel analysis was then conducted using the psych package v.2.4.12 (Revelle, 2024) available in R v.4.4.2 (R Core Team, 2024) to determine the optimal number of factors to retain. The goodness-of-fit of all models was also examined using (e.g., Hu & Bentler, 1999; Marsh et al., 2005; Yu, 2002): the comparative fit index (CFI ≥ 0.90 and >0.95 reflect “*acceptable*” and “*excellent*” fits, respectively), the Tucker–Lewis index (TLI; same thresholds as for the CFI), and the root mean square error of approximation (RMSEA ≤ 0.08 and ≤0.06 represent an “*acceptable*” and “*excellent*” fit, respectively). The composite reliability of the PLSD factor(s) was estimated using the omega (ω) coefficient (McDonald, 1970).

Tests of measurement invariance (equivalence) across country (i.e., Canada vs. France) and sex (i.e., girls vs. boys) was examined in the following sequence (Morin et al., 2011): (a) configural invariance; (b) weak invariance (loadings); (c) strong invariance (loadings and thresholds); (d) strict invariance (loadings, thresholds, and uniquenesses); (e) invariance of the latent variance-covariance; and (f) invariance of latent means. Model comparisons (i.e., each model is contrasted to the previous one) rely on changes (∆) in CFI, TLI, and RMSEA so that invariance is rejected when ∆CFI/∆TLI decreases >0.01 and/or ∆RMSEA increases >0.015 (F. F. Chen, 2007; Cheung & Rensvold, 2002).

Formal tests of measurement invariance are impossible to conduct as a function of naturally continuous variables for which arbitrary dichotomization results in a loss of valuable information (i.e., age, BMI and MVPA in this study). In these situations, it is recommended to assess the impact of these characteristics on item responses and scores on the latent factors through tests of differential item functioning (DIF) implemented using a multiple indicators multiple causes (MIMIC) model. In the present study, separate tests of DIF were performed for age, BMI, and MVPA (time spent in moderate physical activities and in vigorous physical activities were simultaneously included in the same MIMIC model). For each characteristic, these tests involved comparing the following models, in sequence (Marsh et al., 2013; Morin et al., 2013): (a) null effects model (paths from the age, BMI, and MVPA to the latent factors and item responses were constrained to be zero); (b) saturated model (paths from age, BMI, and MVPA to the item responses were freely estimated, while paths from age, BMI, and MVPA to the latent factors were constrained to be zero); and (c) factors-only model (paths from age, BMI, and MVPA to the latent factors were freely estimated, while paths from age, BMI, and MVPA to the item responses were constrained to be zero). Comparing the first two models (null and saturated) tests whether the predictor (age, BMI or MVPA) has an impact on the model, while comparing the last two models (saturated and factors-only) assesses whether this impact is limited to the factors or spreads to item response as well. Model comparisons rely on the same guidelines typically used for tests of measurement invariance, such that ∆CFI and ∆TLI ≥ 0.01 and/or ∆RMSEA ≥ 0.015 are considered meaningful (F. F. Chen, 2007; Cheung & Rensvold, 2002).

Finally, convergent validity was examined using a structural equation model (SEM) in which the PLSD latent factors were correlated with the observed scores (standardized) reflecting parental ratings of children’s overall, cognitive, and motor physical literacy. Importantly, tests of measurement invariance, DIF, and convergent validity were implemented using the exploratory structural equation modeling (ESEM; Morin, 2023) package of Mplus 8.11 (Muthén & Muthén, 2024).

## 3. Results

### 3.1. Exploratory Factor Analysis

**Figure 1:**
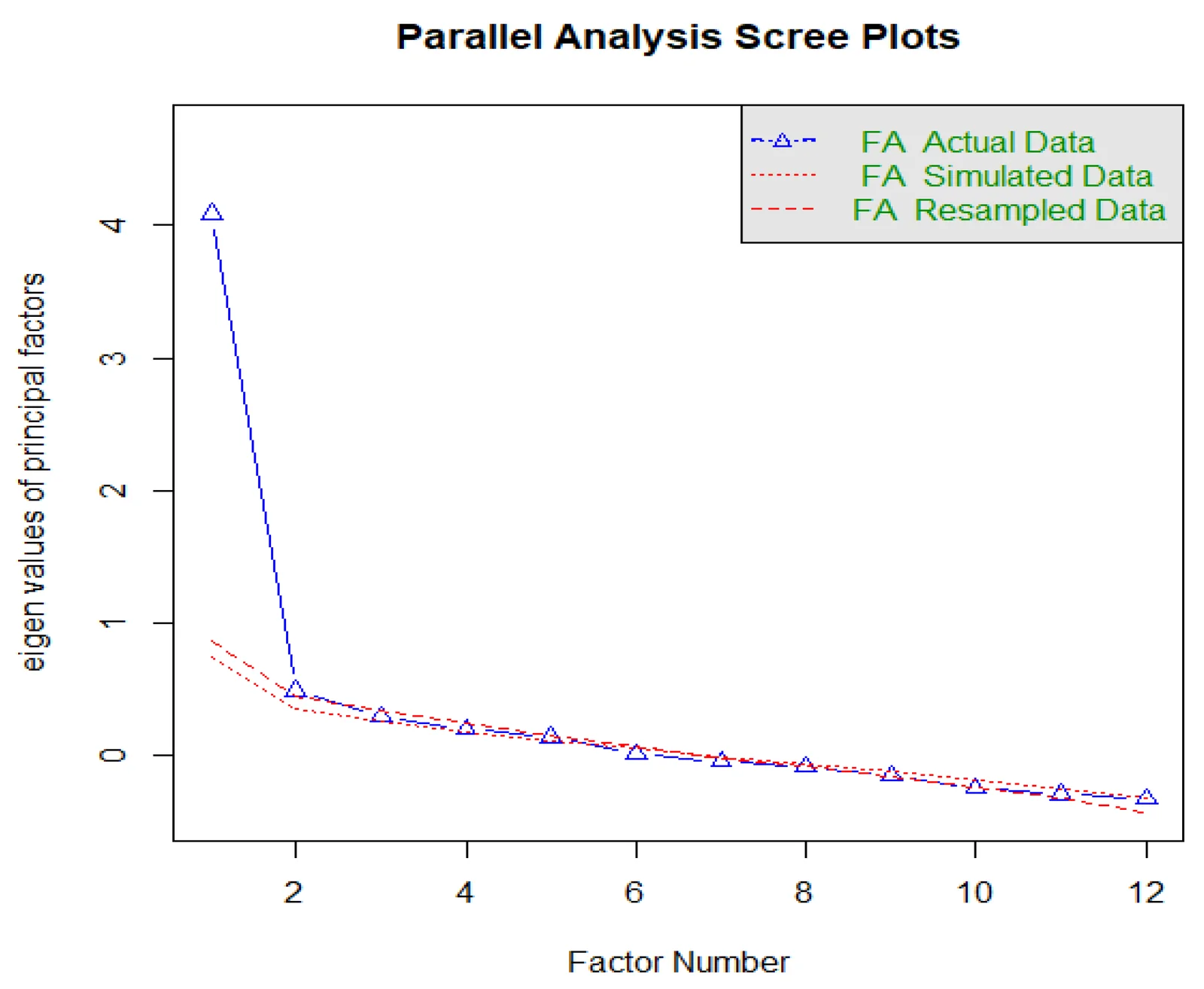
Parallel analysis scree plot from the exploratory factor analysis.

EFA solutions including one to four factors were examined. Model fit indices, reported at the top of Table 1, were acceptable for the unidimensional model and excellent for the two-, three- and four-factor models. Moreover, the parallel analysis test suggested that the data was best represented by a two-factor solution (Figure 1). Parameter estimates from this two-factor solution are reported in Table 2 and reveal that the first factor was primarily defined by items measuring perceived confidence and value (#1, 3, 4, 7, 8, and 9) whereas the second factor was primarily defined by items measuring perceived skills competence (#2, 5, 6, 10, 11 and 12). Although items 5 and 7 showed cross-loadings on both factors, their strongest loadings remained consistent with this interpretation. In contrast, the three-factor solution resulted in the division of the perceived skill competence factor into two three-item factors that were difficult to distinguish conceptually. Likewise, the fourth factor solution further divided the confidence and value factor into separate factors of which one was defined by only a single item. Therefore, although the one-factor model resulted in an acceptable fit to the data, the two-factor solution was retained based on the combined evidence from the parallel analysis, its markedly higher goodness-of-fit indices, and its greater theoretical and substantive interpretability. This two-factor solution was retained for all subsequent psychometric analyses.

As shown in Table 2, the two factors were linked to their main indicators through moderate-to-high factor loadings (perceived confidence and value: λ = −0.346 to 0.699; *M*_λ_ = 0.519; perceived skills competence: λ = 0.356 to 0.694; *M*_λ_ = 0.508). Cross-loadings were generally small (*M*_λ_ = 0.204), although items #5 and 7 cross-loadings were marginally lower than their main loadings, suggesting that these items may be capturing global levels of physical literacy without capturing much specificity related to confidence/value or competence. The latent correlation between the two factors was statistically significant, positive, and modest (i.e., <0.490), whereas composite reliability was satisfactory (ω > 0.700; *M*_ω_ = 0.725).

### 3.2. Measurement Invariance Across Countries and Sex

The fit results of the models used to test the measurement invariance of PLSD responses across countries and sex are, respectively, reported in the second and third sections of Table 1.

#### 3.2.1. Countries

Results support the weak and strong invariance of the model, but not its strict invariance. Examination of the modification indices and parameter estimates associated with these failed invariance solutions indicated that the equality constraint imposed on the residual variance (uniquenesses) of item 11 (“*I’m usually the best in my class at doing an activity*”) represented the primary source of model misfit, as it was substantially higher among French participants than among Canadian participants. This suggested that this item was slightly less reliable in the French sample. Consequently, this equality constraint was removed, resulting in a model of partial strict invariance that provided an adequate fit to the data. However, the invariance of the latent variances, covariance, and means were not supported. As tests of partial invariance of the latent variances, covariances, and means are not possible with ESEM, the previous model of partial strict invariance was thus retained. The results from this model reveal a higher level of inter-individual variability on both factors in Canada than in France. Moreover, scores on both factors were strongly and positively correlated in France (*r* = 0.864, SE = 0.157; *p* < 0.001) but were not significantly related Canada (*r* = −0.440, SE = 0.498; *p* = 0.377). Finally, Canadian children tended to score significantly higher (estimate = 1.010, SE = 0.204; *p* < 0.001) on perceived confidence and value and lower on perceived skills competence (estimate = −0.815, SE = 0.324, *p* = 0.012) than their French peers.

#### 3.2.2. Sex

Results support the weak, strong and strict invariance of the solution. However, the invariance of the latent variances and covariances was rejected. As tests of partial invariance of the latent variances and covariances are not possible with ESEM, the previous model of strict invariance was thus retained, revealing a lower level of inter-individual variability on both factors among boys relative to girls, and a weaker factor correlation among girls (*r* = 0.421, SE = 0.071; *p* < 0.001) relative to boys (*r* = 0.893, SE = 0.217; *p* < 0.001). The final model of latent means invariance was also supported.

### 3.3. DIF Across Age, BMI and MVPA

The fit results of the models used to test the DIF of PLSD responses as a function of age, BMI and MVPA are, respectively, reported in the fourth, fifth and sixth sections of Table 1.

#### 3.3.1. Age

The saturated model had a better fit to the data than the null effects model, revealing a link between age and PLSD responses. Moreover, the fit of the factors-only model was comparable to that of the saturated model, supporting the idea that these associations are limited to the factors (no evidence of DIF). These results indicate that older children tended to score significantly lower on perceived skills competence (*β* = −0.351, SE = 0.092, *p* < 0.001) than younger children.

#### 3.3.2. BMI

Relative to the null effects model, the saturated model showed only a minimal improvement in fit, suggesting that BMI was generally unrelated to participants’ responses. In contrast, the substantial decrease in model fit observed in the factors-only model relative to the saturated model provided evidence of DIF. Further examination of the modification indices, together with the parameter estimates of the saturated model, indicate that DIF was confined to two items. Specifically, higher BMI values were associated with lower scores on item #3 (*I think being active is important for my health and well-being*), whereas higher BMI values were associated with higher scores on item #5 (*I think I can take part in any sport/physical activity that I choose*). Once these effects were added to the factors-only model, the resulting model of partial DIF was supported by the data. Results from this model further showed a lack of relations between BMI and perceived confidence and value (*β* = 0.051, SE = 0.126, *p* = 0.686) or perceived skills competence (*β* = −0.168, SE = 0.121, *p* = 0.165).

#### 3.3.3. MVPA

The saturated model had a better fit to the data than the null effects model, revealing a link between MVPA and PLSD responses. Moreover, the fit of the factors-only model was superior to that of the saturated model, supporting the idea that these associations are limited to the factors (no evidence of DIF). These results indicate that more time spent in moderate physical activity was associated with higher levels of perceived confidence and value (*β* = 0.289, SE = 0.076, *p* < 0.001), whereas more time spent in vigorous physical activity was associated with higher levels of perceived skills competence (*β* = 0.355, SE = 0.098, *p* < 0.001).

### 3.4. Convergent Validity

The fit of the model used to assess convergent validity was excellent [χ^2^(73) = 90.182, CFI = 0.980, TLI = 0.971, RMSEA = 0.035 (90% CI = 0.000, 0.057)]. As reported in Table 3, both PLSD factors were positively related in a statistically significant manner to parental ratings of children’s overall, cognitive and motor physical literacy. Although these associations were modest, they provide evidence of convergent validity while also suggesting that children’s self-perceptions and parents’ evaluations capture related, yet distinct, perspectives on physical literacy.

## 4. Discussion

This study sought to examine the psychometric properties of scores obtained on the French version of the PLSD among French-speaking children recruited in Canada and in France. Previous validation studies focused on the English version of the PLSD yielded inconsistent conclusions in terms of optimal factor structure (Kleis et al., 2022; Lininger & Root, 2024), which the present study was unable to replicate. Rather, the present EFA-based analyses supported a two-factor solution, capturing factors reflecting: (a) perceived confidence and value; and (b) perceived skills competence.

The observation that confidence and value items combined into a single factor is important. Indeed, this result contrasts with those from previous studies conducted among adolescents and emerging adults who identified that these items formed separate factors (Kleis et al., 2022; Lininger & Root, 2024). In contrast, our results suggest that children (French-speaking children in this study, although we expect this difference to be developmental rather than linguistic) may not yet distinguish their beliefs about their ability to participate in physical activity and the value they attribute to such participation. This interpretation is consistent with developmental research showing that physical self-perceptions become increasingly differentiated with age due to cognitive and self-evaluative capacities maturation (e.g., Dreiskämper et al., 2022; Shavelson et al., 1976). Similarly, expectancy-value research indicates that competence beliefs and subjective task values become progressively more distinct across development (e.g., Wigfield & Eccles, 2000). Moreover, in physical literacy conceptualization, confidence and motivation are closely related constructs that support engagement in physical activity (e.g., Whitehead, 2010; Cairney et al., 2019). Therefore, the single dimension measuring comprising confidence- and value-related items may reflect a less differentiated representation of these constructs among children than among older populations.

Conversely, perceived skills competence emerged as a distinct factor. These items focusing on children’s perceptions of their movement and sport-related abilities may be a more concrete and identifiable domain of physical self-assessment. Conversely, the confidence and value factor seems to reflect broader affective and motivational appraisals of participation in physical activity anchored in assessment of specific physical abilities and performance. Therefore, findings from the present study suggest that children seem able to distinguish perceptions of their physical competence from broader motivational and affective perceptions related to physical activity.

Finally, the reversed item #7 (i.e., *I worry about trying a new sport or activity*) displayed weaker psychometric performance than the other items, and its association with the underlying dimensions of the PLSD appeared less clearly defined. One possible explanation is that this item captures aspects of multiple physical literacy domains. For example, worrying about engaging in a new physical activity may reflect not only a lack of confidence, but also lower perceived competence or reduced motivation to participate, thereby limiting its specificity to a single factor. Additionally, negatively worded items are known to introduce measurement artefacts, particularly among younger participants, who may experience greater difficulty processing reverse-coded statements. Age-related variations in the interpretation of this item, as well as contextual differences between the Canadian and French samples, may explain its inconsistent functioning. Future research should examine children’s interpretations of item #7 through qualitative methods (e.g., cognitive interview) and assess its psychometric performance across diverse populations and at various developmental stages.

To date, no evidence was available to inform the possibility of using the PLSD to conduct unbiased comparisons across various subsamples of children. The present study is the first to address this issue, and our results support the possibility of using this instrument for comparisons based on age, MVPA and sex (i.e., no evidence of measurement bias or DIF). We also noted some evidence of measurement bias or DIF as a function of countries and BMI. More precisely, our results show that children’s responses to items #3 (i.e., *I think being active is important for my health and well-being*) and #5 (i.e., *I think I can take part in any sport/physical activity that I choose*) were, respectively, lower and higher for those with a higher BMI, while children’s responses to item #11 (i.e., *I’m usually the best in my class at doing an activity*) were higher in France than in Canada. Although these differences were limited to three out of twelve items, they suggest that caution is warranted when examining group-based differences as a function of children’s countries and BMI based on observed (non-latent) scores. In such situations, it may be preferable to exclude these items or to compare results with and without them, at least. In contrast, latent variable methods, such as those used in this study, allow unbiased comparisons by accounting for partial measurement invariance or DIF.

Additional results revealed significant latent mean differences across countries (i.e., higher levels in perceived confidence and value in Canada; higher levels in perceived skills competence in France), age (lower levels in perceived skills competence in older children) and MVPA (higher levels in perceived confidence and value when children spend more time in moderate physical activity and higher levels of perceived skills competence when they spend more time in vigorous physical activity). The lower levels of perceived skills competence observed among older children are consistent with the idea that children’s self-evaluations may become increasingly realistic as they get older because of increasing reliance on social comparison and cognitive maturity. Nevertheless, this explanation was not directly examined in the present study and should therefore be considered a tentative interpretation requiring further investigation. Furthermore, country-related differences should be interpreted with caution. Although these differences may reflect genuine cross-national variations, the Canadian and French samples also differed in demographic and methodological characteristics (e.g., age, BMI, physical activity levels, recruitment procedures, survey administration methods, and participation incentives). Consequently, the observed differences cannot be attributed solely to cultural or national factors.

In contrast, no latent means differences were found as a function of sex and BMI. Interestingly, we also found meaningful differences in terms of latent variances (revealing more inter-individual variability in Canada and among boys) and covariances (revealing stronger correlations in France and among boys), across grouping variables. Given that this study is the first to assess these differences using the PLSD, some caution is required pending replication to ensure that these differences are more than simple sampling artefacts. Replication in larger and more balanced samples, using harmonized recruitment and data collection procedures across countries, will be necessary to determine the robustness and generalizability of these results and to rule out the possibility that they reflect sampling or methodological artefacts.

Finally, the results support the convergent validity of scores from the French version of the PLSD, as reflected in their associations with parental ratings of children’s overall, cognitive, and motor physical literacy. More specifically, results suggest that parents’ and children’s perceptions were aligned and capture related, yet distinct, perspectives on overall, cognitive and physical competence dimensions of physical literacy.

### Limitations and Directions for Future Research

Several limitations should be acknowledged when considering the findings of the present study. First, the psychometric assessment of the French version of the PLSD was conducted using a single, relatively small sample of French and French-Canadian children. Moreover, participants were recruited using convenience sampling, and no a priori sample size calculation was conducted. Therefore, caution is warranted when generalizing these findings to broader populations of French-speaking youth, to children from other linguistic backgrounds, or to larger and more representative samples. Future research should examine the measurement invariance of PLSD scores across diverse French-speaking contexts, including countries such as Algeria, Belgium, Morocco, Switzerland, and Tunisia, as well as among children speaking other languages, including English, Dutch, Italian, Malay, Portuguese, Spanish, and Turkish.

Second, the factor structure was explored and subsequently evaluated within the same sample, and no independent sample cross-validation was conducted. Consequently, future studies should replicate these findings in independent samples to further establish the robustness, stability and generalizability of the proposed measurement model.

Third, the French subsample was relatively small for the country-specific analyses, which may have reduced the stability and precision of parameter estimates. In addition, children from Canada and France differed on demographic and methodological characteristics. Although measurement invariance analyses supported the comparability of PLSD scores across countries, it remains possible that some of these differences contributed to some of the country-specific findings. Therefore, country-level differences should be interpreted cautiously and considered as preliminary. Future studies relying on larger and more balanced samples, and using harmonized recruitment and data collection procedures, are needed to better isolate country differences and evaluate their robustness.

Fourth, anthropometric data were based on parent-reported height and weight rather than objective assessments. This methodological choice may have introduced some degree of measurement error and could have resulted in lower estimated BMI values than those obtained through direct measurement procedures (Ghosh-Dastidar et al., 2016). Future research should investigate whether our findings extend to studies using directly measured BMI.

Five, the cross-sectional nature of the present study precludes any causal interpretation of associations between time spent in MVPA and physical literacy. Although higher levels of MVPA were associated with more favorable perceptions of physical literacy, it remains unclear whether participation in physical activity contributes to the development of physical literacy, whether children with higher levels of perceived physical literacy are more likely to engage in physical activity, or whether these associations reflect reciprocal influences. Longitudinal studies are needed to clarify the directionality of these relations. In addition, the test–retest reliability and longitudinal invariance of the French version of the PLSD remain to be more thoroughly established through longitudinal research.

Finally, convergent validity was primarily evaluated using parent-reported dimensions of physical literacy, which may limit its scope. Therefore, additional analyses are needed to more comprehensively evaluate its convergent validity in relation to the other *PLAY* tools (Sport for Life, 2022a, 2022b, 2022c, 2022d, 2022e, 2022f), as well as with other children’s test batteries and/or questionnaires developed in Canada (*Canadian Assessment of Physical Literacy* and *Passport for Life*) or elsewhere in the world (e.g., Australia: *Physical Literacy in Children Questionnaire*; China: *Chinese Assessment and Evaluation of Physical Literacy*; and the United Kingdom: *Physical Literacy Charting Tool*).

## 5. Conclusions

The present study revealed that scores on the French version of the PLSD present promising psychometric properties including adequate composite reliability, factor validity, minimal measurement bias and differential item functioning, and evidence of convergent validity. Overall, these findings provide positive and preliminary psychometric evidence supporting the use of the French version of the PLSD among French-speaking children in Canada and France. Additional results suggest the potential suitability of the PLSD for group-based comparisons of physical literacy as a function of the children’s age, BMI, country, MVPA, and sex. However, several limitations of the present study (i.e., sample size, absence of a cross-validation sample, partial support for invariance across countries, DIF observed for BMI, and the possibility that some subgroup findings may reflect sampling variability) suggest that our results should be interpreted as preliminary and replicated in independent and larger samples before firm conclusions can be drawn. Moreover, although some findings point to items that may warrant closer examination, we caution against deleting individual items based solely on the present exploratory and sample-specific results. Finally, the present limitations preclude the use of the French-version of the PLSD in cross-linguistic/cultural and longitudinal studies. Further research is necessary to replicate and extend the present findings.

## Figures and Tables

**Table 1:** Goodness-of-fit statistics for the Physical Literacy Self-Description questionnaire.

Models	N°	Description	Wχ^2^	df	CFI	TLI	RMSEA	RMSEA 90% CI		CM	∆Wχ^2^	df	*p*	∆CFI	∆TLI	∆RMSEA
								LB	UB							
EFA	1-1	1-factor	95.422 *	54	0.951	0.940	0.042	0.063	0.084	-	-	-	-	-	-	-
	1-2	2-factor	63.912	43	0.975	0.962	0.050	0.020	0.075	-	-	-	-	-	-	-
	1-3	3-factor	42.513	33	0.989	0.977	0.039	0.000	0.070	-	-	-	-	-	-	-
	1-4	4-factor	26.801	24	0.997	0.991	0.025	0.000	0.066	-	-	-	-	-	-	-
MI: Country	2-1	Configural invariance	119.686 *	86	0.963	0.944	0.064	0.033	0.090	-	-	-	-	-	-	-
	2-2	Weak invariance	139.584	106	0.964	0.955	0.058	0.026	0.082	2-1	25.656	20	0.178	+0.001	+0.011	−0.007
	2-3	Strong invariance	166.041 *	128	0.959	0.957	0.056	0.027	0.079	2-2	30.123	22	0.111	−0.005	+0.002	−0.002
	2-4	Strict invariance	200.736 *	140	0.934	0.938	0.067	0.045	0.088	2-3	36.354	12	<0.001	−0.025	−0.019	+0.011
	2-5	Partial strict invariance	182.587	139	0.953	0.955	0.057	0.031	0.079	2-3	19.076	11	0.060	−0.006	−0.002	+0.001
	2-6	Variance-covariance invariance	197.079	142	0.940	0.944	0.064	0.040	0.084	2-5	8.677	3	0.034	−0.013	−0.011	+0.007
	2-7	Latent mean invariance	244.467 *	141	0.888	0.895	0.088	0.069	0.106	2-5	66.444	2	<0.001	−0.065	−0.060	+0.031
MI: sex	3-1	Configural invariance	112.440	86	0.975	0.962	0.057	0.019	0.084	-	-	-	-	-	-	-
	3-2	Weak invariance	138.934	106	0.969	0.962	0.057	0.025	0.082	3-1	29.493	20	0.079	−0.006	0.000	0.000
	3-3	Strong invariance	162.011	128	0.968	0.967	0.053	0.021	0.076	3-2	20.698	22	0.540	+0.002	+0.010	−0.008
	3-4	Strict invariance	179.764 *	140	0.963	0.965	0.055	0.026	0.077	3-3	20.234	12	0.063	−0.005	−0.002	+0.002
	3-5	Variance-covariance invariance	260.139 *	143	0.891	0.900	0.093	0.075	0.110	3-4	27.090	3	<0.001	−0.072	−0.065	+0.049
	3-6	Latent mean invariance	171.967	142	0.972	0.974	0.047	0.008	0.070	3-4	0.736	2	0.692	+0.009	+0.009	−0.008
DIF: Age	4-1	Null effects	90.463 *	55	0.961	0.944	0.058	0.035	0.079	-	-	-	-	-	-	-
	4-2	Saturated	64.424	43	0.976	0.957	0.051	0.021	0.076	4-1	24.727	12	0.016	+0.015	+0.013	−0.007
	4-3	Factors-only	82.705 *	53	0.967	0.952	0.054	0.030	0.076	4-1	5.063	2	0.080	+0.006	+0.008	−0.004
DIF: BMI	5-1	Null effects	78.043	55	0.973	0.962	0.047	0.018	0.069	-	-	-	-	-	-	-
	5-2	Saturated	61.110	43	0.979	0.962	0.047	0.013	0.072	5-1	18.633	12	0.010	+0.006	0.000	0.000
	5-3	Factors-only	83.308 *	53	0.965	0.948	0.055	0.030	0.076	5-1	1.690	2	0.430	−0.008	−0.014	+0.008
	5-4	Partial DIF	73.539	51	0.974	0.960	0.048	0.019	0.071	5-2	13.289	8	0.102	−0.005	−0.002	+0.001
DIF: MVPA	6-1	Null effects	118.545 *	67	0.941	0.920	0.063	0.044	0.082	-	-	-	-	-	-	-
	6-2	Saturated	62.526	43	0.978	0.953	0.049	0.017	0.074	6-1	54.580	24	<0.001	+0.037	+0.033	−0.014
	6-3	Factors-only	75.197	63	0.986	0.980	0.032	0.000	0.056	6-1	20.752	4	<0.001	+0.045	+0.060	−0.031

Note. Wχ^2^ = robust weighed least square (WLSMV) chi-square; df = degrees of freedom; CFI = comparative fit index; TLI = Tucker–Lewis index; RMSEA = root mean square error of approximation; 90% CI = 90% confidence interval of the RMSEA; CM = comparison model; LB = lower bound; UB = upper bound; ∆Wχ^2^ = WLSMV chi square difference test (calculated with the Mplus DIFFTEST function); ∆ = change from the previous model; EFA = exploratory factor analysis; MI = measurement invariance; DIF = differential item functioning; BMI = body mass index; MVPA = moderate-to-vigorous physical activities. * *p* ≤ 0.01.

**Table 2:** Standardized parameter estimates from the exploratory structural equation model of the Physical Literacy Self-Description questionnaire.

Items	Perceived Confidence and Value (λ)	Perceived Skills Competence (λ)	δ
1	**0.580**	0.291	0.414
2	*0.132*	**0.601**	0.545
3	**0.699**	*0.002*	0.509
4	**0.529**	*0.109*	0.652
5	0.342	**0.356**	0.638
6	0.239	**0.490**	0.588
7	**−0.346**	0.333	0.882
8	**0.506**	*0.098*	0.685
9	**0.455**	0.328	0.540
10	0.315	**0.468**	0.538
11	*−0.054*	**0.694**	0.552
12	0.201	**0.440**	0.680
ω	0.725	0.724	
Perceived Skills Competence	0.488	-	

Note. λ = factor loadings; δ = uniquenesses; ω = McDonald’s omega. Main loadings are in bold. Non-significant loadings are underlined and italicized.

**Table 3:** Convergent validity from the 2-factor exploratory factor analytic representation of the Physical Literacy Self-Description questionnaire.

Parents’ Assessment of Children’s	Perceived Confidence and Value	Perceived Skills Competence
Overall level of physical literacy	0.391 **	0.319 **
Cognitive dimension of physical literacy	0.356 **	0.328 **
Motor competence dimension of physical literacy	0.288 **	0.194 *

Note. * *p* ≤ 0.05; ** *p* ≤ 0.01.

## Data Availability

The data is unavailable due to ethical restrictions.

## Note

1 It remains important to also acknowledge international developments, such as the *Chinese Assessment and Evaluation of Physical Literacy* (China: S. T. Chen et al., 2020), *Physical Literacy Charting Tool* (United Kingdom; International Physical Literacy Association, 2018), and *Physical Literacy in Children Questionnaire* (Australia: Barnett et al., 2022).

## Abbreviations

The following abbreviations are used in this manuscript:

PLSD	Physical Literacy Self-Description
PCA	Principal component analysis
EFA	Exploratory factor analysis
BMI	Body mass index
VAS	Visual analog scale
CLASS	Children’s Leisure Activities Study Survey
MVPA	Moderate-to-vigorous physical activities
WLSMV	Robust weighted least squares with mean and variance adjustment
CFI	Comparative fit index
TLI	Tucker–Lewis index
RMSEA	Root mean square error of approximation
DIF	Differential item functioning
MIMIC	Multiple indicators multiple causes
ESEM	Exploratory structural equation modeling
RMSEA 90% CI	90% confidence interval of the RMSEA
CM	Comparison model
